# Thresholds for meaningful change in Mini-Mental State Examination scores in rare dementias

**DOI:** 10.1186/s13195-026-02136-y

**Published:** 2026-07-11

**Authors:** Tamar Abzhandadze, Minh Tuan Hoang, Xinqi Bao, Madeleine Åkerman, Jakob Norgren, Belén García Pascual, Minjia Mo, Maria Eriksdotter, Hong Xu, Sara Garcia-Ptacek

**Affiliations:** 1 Department of Neurobiology, Care Sciences and Society (NVS), Division of Clinical Geriatrics, Karolinska Institutet, Stockholm, Sweden; 2 Department of Occupational Therapy and Physiotherapy, Sahlgrenska University Hospital, Gothenburg, Sweden; 3 Faculty of Public Health, University of Medicine and Pharmacy, Vietnam National University, Hanoi, Vietnam; 4 Department of Intelligent Systems, KTH Royal Institute of Technology, Stockholm, Sweden; 5 Department of Mathematics, KTH Royal Institute of Technology, Stockholm, Sweden; 6 Aging and Inflammation Theme, Karolinska University Hospital, Stockholm, Sweden

**Keywords:** Assessment, Cognition, Diagnosis, Disease, Frontotemporal Dementia, Lewy Body, Minimal clinically important difference, Minimal detectable change, Mini-Mental State Examination

## Abstract

**Background:**

We conceptualize the Real-World Reassessment Threshold (RWRT) as representing the smallest change that exceeds expected measurement variability while accounting for clinically expected cognitive decline over the assessment interval, whereas the minimum clinically important difference (MCID) indicates the smallest change likely to be clinically meaningful. To date, no study has empirically defined the RWRT or MCID for Mini-Mental State Examination (MMSE) scores in Lewy body dementia (LBD) or frontotemporal dementia (FTD), limiting the interpretation of longitudinal changes and clinical trial designs. We therefore aimed to estimate 12-month, diagnosis-specific MMSE thresholds for RWRT and MCID among individuals with LBD and FTD, and to evaluate the generalizability of these thresholds in an independent validation cohort.

**Methods:**

This registry-based cohort study included individuals diagnosed with LBD or FTD from the Swedish Registry for Cognitive/Dementia Disorders (SveDem, 2007–2022) with a 91–400-day MMSE follow-up, and an independent validation cohort from the U.S. National Alzheimer’s Coordinating Center (NACC). The RWRT was estimated using distribution-based methods based on intraclass correlation coefficients (ICCs). MCID was estimated using both anchor-based and distribution-based (0.5 standard deviation) approaches.

**Results:**

We included 1,158 individuals from SveDem (873 LBD, 285 FTD) and 1,060 individuals from NACC (469 LBD, 591 FTD). Over the 91–400-day follow-up interval, MMSE scores demonstrated moderate to high reliability (ICC 0.70–0.90), corresponding to RWRT estimates ranged from 5 to 7 MMSE points. Anchor-based MCIDs differed by diagnosis, with a mean threshold of 0.7 points in LBD and 3.8 points in FTD in SveDem; similar diagnosis- and baseline severity-dependent patterns were observed in NACC. In contrast, distribution-based MCIDs were consistent across diagnoses, clustering around 2–3 MMSE points.

**Conclusions:**

MMSE changes of less than five points over one year may reflect expected test variability combined with expected individual decline. However, average changes of 2–3 points may still be meaningful when combined with a clinical anchor of change, depending on diagnosis and disease stage. These results emphasize the importance of using both RWRT and MCID when evaluating MMSE change and selecting clinical trial endpoints.

**Supplementary Information:**

The online version contains supplementary material available at 10.1186/s13195-026-02136-y.

## Background

Lewy body dementia (LBD, including both Dementia with Lewy bodies [DLB] and Parkinson’s disease dementia [PDD]) and Frontotemporal dementia (FTD) are common non-Alzheimer’s dementias that place a substantial burden on patients and caregivers [1, 2]. The Mini-Mental State Examination (MMSE) is widely used in both routine clinical care and research settings to assess global cognition owing to its familiarity, ease of administration, and cross-study comparability [3–5]. However, changes in MMSE scores are often interpreted without diagnosis-specific thresholds to distinguish true cognitive decline from expected variability in repeated testing or to determine whether a change is clinically meaningful. This limitation is particularly pronounced in LBD, where attentional fluctuations, visuospatial deficits, and motor or visual interference introduce considerable measurement variability, and in FTD, where executive dysfunction, language impairment, behavioral symptoms, and social-cognitive deficits are inadequately captured by the MMSE [6].

To date, no study has empirically defined the real-world reassessment threshold (RWRT) or the minimum clinically important difference (MCID) for the MMSE in LBD or FTD. We define RWRT as the smallest change that exceeds expected measurement variability while accounting for clinically expected cognitive decline over the assessment interval, whereas the MCID is the smallest MMSE change likely to be clinically meaningful. These thresholds are important in dementia research and clinical practice because they can help determine whether an observed MMSE change reflects clinically meaningful change, expected decline, or variability in repeated testing.

The annual rate of MMSE decline in LBD averages approximately 2.0–4.4 points per year, similar to or slightly faster than that observed in Alzheimer’s disease (AD; ~ 2.0–3.0 points/year), whereas FTD exhibits a broader, phenotype-dependent range of decline (~ 0.9–6.7 points/year) [7–14]. Despite these differences, thresholds of meaningful change are typically extrapolated from AD cohorts, such as the widely cited 1–2 point annual decline, which may not accurately reflect disease-specific progression in LBD or FTD [15].

The absence of empirically derived thresholds limits the interpretability of MMSE score changes and hampers the ability to distinguish true cognitive decline from normal variability or to guide clinical and therapeutic decision-making [16]. Therefore, we aimed to derive 12-month, diagnosis-specific MMSE thresholds for detecting statistically (RWRT) and clinically (MCID) meaningful changes in LBD and FTD, and to evaluate their generalizability using external validation in an independent cohort.

## Methods

### Design and setting

This cohort study was conducted and reported in accordance with the Strengthening the Reporting of Observational Studies in Epidemiology guidelines [17]. The study cohort was drawn from the Swedish Registry for Cognitive/Dementia Disorders (SveDem), using baseline and follow-up modules [18]. An independent validation cohort was obtained from the National Alzheimer’s Coordinating Center (NACC) to ensure external validity [19]. Variables from the NACC Uniform Data Set (UDS) were used, including the Header and Milestones Form, Subject Demographics Form (FORM A1), Neuropsychological Battery Summary Score Forms (Forms C1, C2, and C2T), and the Clinical Dementia Rating (CDR)® Dementia Staging Instrument Plus NACC FTLD Behavior & Language Domains (CDR® Plus NACC FTLD [Form B4]) [19].

This study utilized several nationwide data sources, linked using the unique Swedish personal identity number, enabling identification of individuals with dementia and integration of clinical, demographic, and prescription data. The primary dataset, SveDem [18, 20], is a national quality registry established in 2007 to improve dementia care in Sweden. SveDem records diagnostic procedures, medical treatments, and community support services, including home-based and institutional care, with annual follow-ups conducted in memory clinics, primary care centers, and nursing homes. From SveDem, we extracted MMSE scores, clinician-rated global impressions of functional change, and sociodemographic variables. Educational attainment was obtained from the Longitudinal Integration Database for Health Insurance and Labour Market Studies.

The external validation cohort was obtained from the NACC database, which compiles standardized longitudinal data from Alzheimer’s Disease Research Centers (ADRCs) across the United States using the UDS protocol [19]. UDS collects harmonized demographic, clinical, neurological, and neuropsychological data at regular intervals, typically annually, through in-person, home, or remote assessments, with Milestone Forms documenting major changes such as relocation, death, or study discontinuation. Diagnoses are assigned by either a consensus panel or the evaluating physician, depending on ADRC practice. Although focused on AD, ADRCs also enroll individuals with other types of dementia [19]. From NACC, we retrieved variables aligned a priori with SveDem, including MMSE, demographic characteristics, and follow-up interval, to maximize cross-cohort comparability.

### Study sample

The index date for each individual was defined as the date of the initial dementia diagnosis recorded in SveDem between 2007 and 2022. Clinician-assigned diagnoses, as recorded in SveDem, were retrieved and categorized as LBD (including DLB and PDD) and FTD. Data on diagnoses were retrieved from NACC for the period 2005–2024, and participants were classified according to their primary NACC-assigned diagnosis of LBD or FTD, including all available FTD subtypes [21]. Individuals were required to have MMSE data available at baseline and at follow-up within a prespecified interval of 91–400 days between assessments. The 91–400-day interval was selected to approximate routine annual follow-up in real-world dementia care while allowing for scheduling variability. Therefore, the resulting RWRT estimates should be interpreted as reliable-change thresholds over an annual clinical follow-up interval, rather than as short-term test–retest measurement-error estimates. Applying identical criteria to the NACC cohort ensured methodological consistency across datasets.

### Variables

Cognitive function was assessed with the MMSE [22], administered by trained personnel following standardized procedures. The MMSE is a brief cognitive screening instrument scored from 0 to 30. Based on clinical consensus among the authors, baseline MMSE scores were categorized into three strata (≥ 26, 20–25, and ≤ 19). In addition, we conducted an analysis restricted to individuals with mid-range MMSE scores (15–27). This range approximates the mid-score region in which the MMSE exhibits relatively greater sensitivity to change than scores near the upper (27–30) or lower extremes [23].

The MMSE data were retrieved at baseline and at a follow-up between 91 and 400 days. If multiple follow-up assessments occurred within this window, we used the earliest assessment to minimize regression-to-the-mean effects. In SveDem, annual follow-up is defined as approximately three months after the last registration. The upper bound of 400 days, approximately one month beyond a full year, was selected to accommodate variability in care schedules and practical differences in follow-up timing.

The global impression of functional change was used as an anchor in the MCID analysis [24]. This variable, systematically collected at each follow-up visit in SveDem, captures participants’ overall capabilities and everyday functioning. The global impression of functional change was categorized as unchanged, worsened, or improved, with the worsened and improved categories combined into a single group representing any clinically meaningful change, consistent with anchor-based MCID methodology. For the NACC cohort, diagnostic categories were matched using available NACC diagnosis codes, and MMSE data were collected using standardized protocols. As NACC does not include a direct equivalent of the global impression measure used in SveDem, the CDR scale was used as a proxy [21, 25, 26]. The CDR is a clinician-rated instrument that evaluates cognitive and functional performance across six domains—memory, orientation, judgment and problem-solving, community affairs, home and hobbies, and personal care, and produces a global score ranging from 0 to 3, reflecting overall dementia severity [25]. A change of > 0.5 points in the global CDR score was considered to reflect clinically meaningful progression [26], distinguishing true change from minor fluctuations. Because SveDem and NACC do not capture identical functional-change anchors, anchor-based MCID was derived separately for each cohort, with cross-cohort consistency interpreted primarily in terms of directionality and relative patterns across baseline severity strata.

Both the study cohort and the validation cohort were described in terms of sex, age at diagnosis (dichotomized at ≤ 74 vs. ≥ 75 years), number of medications used before diagnosis, cohabitation status, and accommodation type.

### Statistical analysis

For clinical interpretation, RWRT (real-world reassessment threshold) and MCID address related but distinct questions. RWRT estimates whether an observed MMSE change exceeds both measurement variability and expected time-related cognitive decline, whereas MCID estimates whether an observed change is clinically meaningful. Given the 91–400-day follow-up interval, traditional MDC terminology was not appropriate; therefore, we introduced the term RWRT to denote an interval-specific, real-world reliable-change threshold.

Primary analyses estimated RWRT and MCID for LBD and FTD in SveDem, with external validation in the NACC cohort. Analyses were harmonized across datasets, diagnostic groups, and subgroups. The study sample was described using n (%) for categorical variables and mean (SD) for continuous variables, with 95% CIs reported due to small subgroup sizes. Bland–Altman plots assessed agreement between baseline and follow-up MMSE scores, with mean differences and 95% limits of agreement (LoA) calculated for each diagnostic group.

The RWRT at the 95% confidence level was estimated using the intraclass correlation coefficient (ICC) [27–29], based on baseline and follow-up MMSE scores collected 91–400 days apart. The ICC quantified the reliability of repeated MMSE measurements: higher ICC values indicate greater stability and lower measurement variability, whereas lower ICC values indicate greater variability. In the MDC formula, the ICC was used to estimate the standard error of measurement; therefore, lower reliability produced a larger RWRT [29]. Analyses were conducted under three conditions: (i) restricting the sample to individuals whose MMSE change fell within ± 2 SD to reduce the influence of outliers [30]; (ii) using the full range of baseline MMSE scores; and (iii) restricting the sample to participants with baseline MMSE scores between 15 and 27. RWRT was calculated using the standard MDC formula as follows [30]:

$${MDC}_{95}=\left({SD}_{pooled}\times \sqrt{1-ICC}\right)\times \left(1.96\times \sqrt{2}\right)$$

where,

$${SD}_{pooled}=\sqrt{\frac{SD {baseline}^{2}+SD follow-{up}^{2}}{2}}$$

SD_pooled_ is the pooled SD of repeated MMSE scores. The term √(1 − ICC) converts the pooled SD into the standard error of measurement. The multiplier 1.96 reflects the 95% confidence level, and √2 accounts for uncertainty from two MMSE measurements: baseline and follow-up. The ICC was calculated using a two-way random-effects model for absolute agreement based on baseline and follow-up MMSE scores.

MCID was estimated using both anchor- and distribution-based approaches. For the anchor-based method, changes in MMSE scores were linked to clinician-rated global impression of functional change in SveDem, and to changes in the global CDR score in NACC, with worsening or improvement > 0.5 points considered clinically meaningful [26]. The > 0.5-point threshold was chosen because a change of this magnitude represents movement beyond a stable global severity category and has commonly been used to indicate clinically meaningful change on the global dementia rating scale [26]. Anchor-based MCID values are presented as absolute mean MMSE changes; therefore, larger values indicate greater magnitude of clinically meaningful change, regardless of direction. Distribution-based MCID was derived using 0.5 SD of baseline MMSE scores [31]. Analyses were stratified by baseline age group (categorized as ≤ 74 and ≥ 75 years), cognitive function at baseline (MMSE scores ≥ 26, 20–25, and ≤ 19), as well as by the mid-range MMSE stratum (15–27).

Sensitivity analyses were conducted within the LBD group in SveDem, using the same methods as the primary RWRT and MCID calculations, but stratified by DLB and PDD subgroups.

No imputation was performed; the number (%) of missing values for each variable was reported. All analyses were performed using RStudio (version 2024.4.1.748) and IBM SPSS Statistics (Version 30; IBM Corp., Armonk, NY, USA). All statistical tests were two-sided, at alpha 5%.

## Results

### Study sample

Between 2007 and 2022, 5,935 individuals in SveDem were diagnosed with LBD (DLB or PDD) or FTD (Supplementary Fig. 1). Of these, 4,777 were excluded owing to the absence of follow-up data, follow-up intervals outside the 91–400-day window, or missing MMSE data at baseline or follow-up. The included individuals (*n* = 1,158) were younger and more frequently male than those excluded. The median age of included individuals was 74 years (IQR 9) versus 75 years (IQR 10) among those excluded (*p* < 0.001). Men accounted for 64% of included individuals compared with 58% of those excluded (*p* < 0.001).

The study sample comprised 1,158 individuals, of whom 873 had LBD (mean age 74.9 [SD 6.5] years, 32% women). The validation cohort comprised 1,060 individuals, of whom 469 had LBD (mean age 72.9 [SD 8.4] years, 22% women). Characteristics of the study and validation samples are shown in Table 1.

**Table 1:** Baseline characteristics of individuals with LBD and FTD in SveDem and NACC

	SveDem, *n* = 1,158				NACC, *n* = 1,060			
	LBD, *n* = 873		FTD, *n* = 285		LBD, *n* = 469		FTD, *n* = 591	
Variables/categories	Mean (SD) or n (%)	95% CI	Mean (SD) or n (%)	95% CI	Mean (SD) or n (%)	95% CI	Mean (SD) or n (%)	95% CI
Age at diagnosis, years	74.9 (6.5)	74.5, 75.5	70.2 (7.9)	69.3, 71.1	72.9 (8.4)	72.1, 73.7	65.8 (9.8)	65.0, 66.6
**Sex**								
Male	593 (67.9)	64.8, 71.0	148 (51.9)	46.1, 57.7	365 (77.8)	73.9, 81.4	348 (58.9)	54.9, 62.8
Female	280 (32.1)	29.0, 35.2	137 (48.1)	42.3, 53.9	104 (22.2)	18.6, 26.1	243 (41.1)	37.2, 45.1
**Education**								
Primary or below (≤ 9 y)	80 (22.9)	18.7, 27.5	32 (23.5)	17.0, 31.2	15 (3.2)	1.9, 5.1	12 (2.0)	1.1, 3.4
Secondary (10‒12 y)	145 (41.5)	36.5, 46.8	70 (51.5)	43.1, 59.8	89 (19.0)	15.6, 22.7	146 (24.7)	21.4, 28.3
University or above (≥ 13 y)	124 (35.5)	30.6, 40.7	34 (25.0)	18.3, 32.8	365 (77.8)	73.9, 81.4	433 (73.3)	69.6, 76.7
**Marital status**								
Married/cohabiting	231 (66.0)	60.9, 70.8	79 (57.7)	49.3, 65.7	394 (84.4)	80.9, 87.4	493 (83.8)	80.7, 86.6
Widowed	34 (9.7)	6.9, 13.1	11 (8.0)	4.3, 13.5	43 (9.2)	6.8, 12.1	38 (6.5)	4.7, 8.7
Single/divorced	85 (24.3)	20.0, 29.0	47 (34.3)	26.8, 42.5	30 (6.4)	4.5, 8.9	57 (9.7)	7.5, 12.3
**Cohabitation status**								
Living alone	166 (22.5)	19.6, 25.6	66 (26.6)	21.4, 32.4	43 (9.2)	6.8, 12.1	57 (9.7)	7.5, 12.2
Cohabiting/other	573 (77.5)	74.4, 80.4	182 (73.4)	67.6, 78.6	425 (90.8)	87.9, 93.2	533 (90.3)	87.8, 92.5
**Accommodation**								
Ordinary housing	762 (87.7)	85.4, 89.7	266 (93.3)	90.0, 95.8	425 (93.0)	90.4, 95.1	540 (92.6)	90.3, 94.5
Institutional care	107 (12.3)	10.3, 14.6	19 (6.7)	4.2, 10.0	32 (7.0)	4.9, 9.6	43 (7.4)	5.5, 9.7
**Baseline cognition**								
MMSE ≥ 26	200 (22.9)	20.2, 25.8	69 (24.2)	19.5, 29.4	265 (56.5)	52.0, 60.9	267 (45.2)	41.2, 49.2
MMSE 20–25	423 (48.5)	45.1, 51.8	125 (43.9)	38.2, 49.7	144 (30.7)	26.7, 35.0	184 (31.1)	27.5, 35.0
MMSE ≤ 19	250 (28.6)	25.7, 31.7	91 (31.9)	26.7, 37.5	60 (12.8)	10.0, 16.0	140 (23.7)	20.4, 27.2

Variables with missing data n (%): In SveDem, variables with missing data were: accommodation, *n* = 4 (0.3%); cohabitation status, *n* = 171 (14.8%); education, *n* = 673 (58.1%); and marital status, *n* = 671 (57.9%). In NACC missing data were present for marital status, *n* = 5 (0.5%); cohabitation status, *n* = 2 (0.2%); accommodation, *n* = 20 (1.9%). Percentages were calculated using non-missing data for each variable

*Abbreviations*: *MMSE* Mini-Mental State Examination, *SD* standard deviation, *95% CI* 95% confidence interval, *SveDem* the Swedish Registry for Cognitive/Dementia Disorders, *NACC* the National Alzheimer’s Coordinating Center, *LBD* Lewy body dementia, including both dementia with Lewy bodies (DLB) and Parkinson’s disease dementia (PDD), *FTD* frontotemporal dementia

### RWRT of the MMSE

**Fig. 1:**
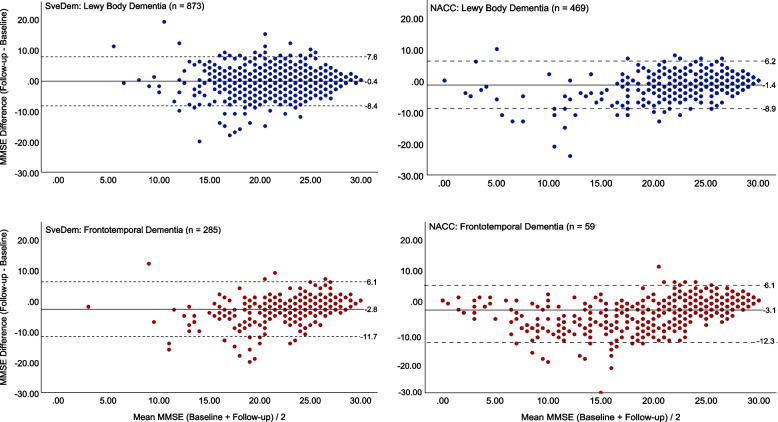
Bland–Altman plots of MMSE change in LBD and FTD (SveDem and NACC). The x-axis shows mean MMSE ([baseline + follow-up]/2), and the y-axis shows the score difference. Solid lines indicate the mean; dashed lines show 95% limits of agreement. MMSE, Mini-Mental State Examination; SveDem, the Swedish Registry for Cognitive/Dementia Disorders; NACC, the National Alzheimer’s Coordinating Center; LBD, Lewy body dementia; FTD, frontotemporal dementia

The RWRT reflects whether an observed change exceeds what would be expected from real-world retest variability combined with interval-specific disease-related decline. All diagnostic groups showed small negative mean differences between baseline and follow-up (Fig. 1). LBD displayed moderate variability, whereas FTD, particularly in the NACC cohort, showed wider LoA, reflecting greater heterogeneity in MMSE change (Fig. 1). In SveDem, baseline and 91–400-day MMSE test–retest reliability was moderate to good within the MMSE ± 2 SD group, with ICCs of 0.8 (95% CI, 0.8‒0.8) in LBD and 0.8 (95% CI, 0.8‒0.9) in FTD, corresponding to RWRTs of 5.1 and 4.9 points, respectively. Across MMSE strata (15–27 and the full score range), ICCs remained between 0.7 and 0.8, and RWRTs remained between 5.1 and 7.1 points, indicating that changes smaller than these thresholds may not be distinguishable from expected real-world variability over the 91–400-day follow-up interval (Table 2) without a clinical anchor. These results were supported in the NACC cohort, where ICCs were slightly higher for both LBD and FTD within the MMSE ± 2 SD group, with comparable RWRT magnitudes of 4.1–6.8 points across diagnostic groups and MMSE strata (Table 2).

**Table 2:** RWRT of MMSE among individuals with LBD and FTD in SveDem and NACC

	SveDem, *n* = 1,158		NACC, *n* = 1,060	
	LBD	FTD	LBD	FTD
***MMSE,* ± *2 SD***	*n* = *841*	*n* = *262*	*n* = *428*	*n* = *529*
MMSE_baseline,_ mean (95% CI)	22.3 (22.0, 22.6)	24.0 (23.6, 24.5)	25.0 (24.5, 25.5)	22.8 (22.3, 23.4)
MMSE_follow-up,_ mean (95% CI)	22.0 (21.7, 22.3)	22.1 (21.5, 22.6)	24.2 (23.7, 24.7)	20.8 (20.2, 21.5)
SD_mmse_baseline_	4.1	3.9	5.0	6.8
SD_mmse_follow-up_	4.3	4.8	5.6	8.0
SD_pooled_	4.2	4.4	5.3	7.4
ICC (95% CI)	0.8 (0.8, 0.8)	0.8 (0.8, 0.9)	0.9 (0.9, 0.9)	0.9 (0.9, 1.0)
RWRT	5.1	4.9	4.1	4.6
***MMSE 15–27 at baseline***	*n* = *730*	*n* = *205*	*n* = *253*	*n* = *333*
MMSE_baseline,_ mean (95% CI)	22.2 (22.0, 22.4)	23.1 (22.6, 23.5)	23.4 (23.1, 23.8)	23.2 (22.8, 23.6)
MMSE_follow-up,_ mean (95% CI)	21.9 (21.6, 22.2)	21.3 (20.7, 21.9)	22.9 (22.4, 23.4)	21.2 (20.6, 21.7)
SD_mmse_baseline_	3.2	3.0	3.1	3.3
SD_mmse_follow-up_	4.0	4.3	4.4	5.4
SD_pooled_	3.6	3.7	3.8	4.5
ICC (95% CI)	0.7 (0.7, 0.8)	0.8 (0.7, 0.8)	0.8 (0.7, 0.8)	0.8 (0.8, 0.9)
RWRT	5.2	5.1	5.0	5.3
***All MMSE scores***	*n* = *873*	*n* = *285*	*n* = *469*	*n* = *591*
MMSE_baseline,_ mean (95% CI)	22.2 (21.9, 22.5)	24.0 (23.6, 24.5)	24.7 (24.3, 25.2)	22.7 (22.2, 23.3)
MMSE_follow-up,_ mean (95% CI)	21.8 (21.5, 22.1)	21.2 (20.6, 21.9)	23.4 (22.8, 23.9)	19.6 (19.0, 20.3)
SD_mmse_baseline_	4.3	4.1	5.2	6.6
SD_mmse_follow-up_	4.6	5.5	6.6	8.5
SD_pooled_	4.4	4.9	5.9	7.6
ICC (95% CI)	0.7 (0.7, 0.8)	0.7 (0.7, 0.8)	0.9 (0.9, 0.9)	0.9 (0.9, 0.9)
RWRT	6.4	7.1	5.7	6.8

*Abbreviations*: *MMSE* Mini-Mental State Examination, *SD* standard deviation, *ICC (95% CI)* intraclass correlation coefficient with 95% confidence interval from a two-way random-effects model, *RWRT* real-world reassessment threshold at the 95% confidence level, *SveDem* the Swedish Registry for Cognitive/Dementia Disorders, *NACC* the National Alzheimer’s Coordinating Center. *LBD* Lewy body dementia, including both dementia with Lewy bodies (DLB) and Parkinson’s disease dementia (PDD), *FTD* frontotemporal dementia

### MCID of the MMSE

**Fig. 2:**
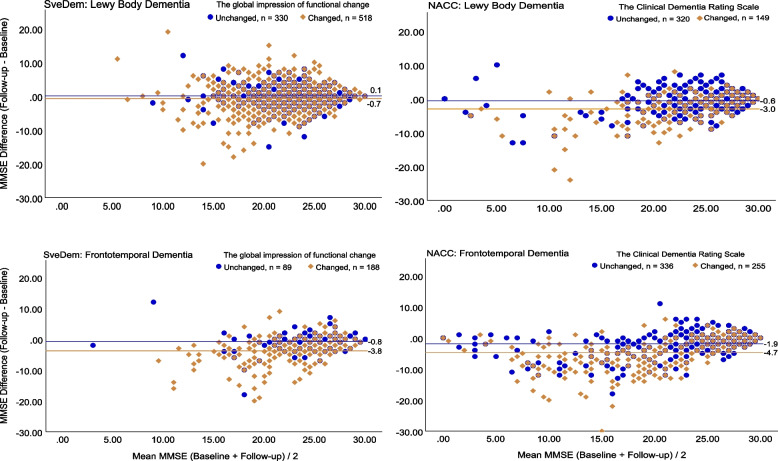
Bland–Altman plots of MMSE change in LBD and FTD (SveDem and NACC). The x-axis shows mean MMSE ([baseline + follow-up]/2), and the y-axis shows the score difference; the solid line indicates the mean. Analyses are shown for each diagnostic group, stratified by the global impression of functional change (for SveDem) and the Clinical Dementia Rating scale (NACC). MMSE, Mini-Mental State Examination; SveDem, the Swedish Registry for Cognitive/Dementia Disorders; NACC, the National Alzheimer’s Coordinating Center; LBD, Lewy body dementia; FTD, frontotemporal dementia

Distribution-based MCID indicates whether the magnitude of change is meaningful relative to the statistical properties and variability of the measure, whereas anchor-based MCID relates observed change to an external clinical criterion. Across cohorts and diagnoses, individuals classified as functionally changed showed greater MMSE decline (~ 3 to 5 points) than those rated unchanged (≈ 0 to 1 point), with consistently greater variability observed in the changed group (Fig. 2).

In SveDem, the anchor-based MCID was small for LBD overall (0.7 points; 95% CI, 0.3–1.1) but markedly larger for FTD (3.8 points; 95% CI, 3.1‒4.4) (Table 3). In both groups, anchor-based MCID increased with greater cognitive impairment, most prominently in FTD (up to 6.8 points; 95% CI, 5.7‒7.8 for MMSE ≤ 19), whereas distribution-based MCIDs were more homogeneous, clustering around approximately 2 MMSE points across age and MMSE strata (range ~ 1.2–2.4).

**Table 3:** MCID for the MMSE in SveDem across diagnoses, age, and cognitive function at baseline. Anchor-based MCID values are presented as absolute mean MMSE changes; therefore, larger values indicate greater magnitude of clinically meaningful change, regardless of direction

Diagnosis	N, completed	Change in global status, n (%)	Anchor-based MCID, absolute mean change (95% CI)	Distribution-based MCID
**LBD**				
All individuals	873	518 (59.3)	0.7 (0.3, 1.1)	2.1
*Age groups*				
≤ 74 y	397	232 (58.4)	0.7 (0.1, 1.2)	2.2
≥ 75 y	476	286 (60.1)	0.8 (0.2, 1.3)	2.1
*Baseline cognition*				
MMSE 15–27	748	433 (57.9)	0.5 (0.2, 0.9)	2.0
MMSE ≥ 26	200	101 (50.5)	2.8 (2.2, 3.5)	1.5
MMSE 20–25	423	237 (56.0)	0.3 (0.2, 0.8)	1.7
MMSE ≤ 19	250	180 (72.0)	3.3 (2.6, 4.0)	2.2
**FTD**				
All individuals	285	188 (66.0)	3.8 (3.1, 4.4)	2.0
Age groups				
≤ 74 y	198	140 (70.7)	3.9 (3.1, 4.7)	2.0
≥ 75 y	87	48 (55.2)	3.4 (2.1, 4.6)	2.2
*Baseline cognition*				
MMSE 15–27	214	139 (65.0)	3.2 (2.6, 3.8)	1.8
MMSE ≥ 26	69	38 (55.1)	0.6 (0.3, 1.5)	1.2
MMSE 20–25	125	74 (59.2)	2.9 (2.3, 3.6)	1.4
MMSE ≤ 19	91	76 (83.5)	6.8 (5.7, 7.8)	2.4

For anchor-based MCID analyses, N completed refers to individuals with available MMSE change and non-missing global impression of functional change. Therefore, subgroup Ns may differ from those used in RWRT analyses

*Abbreviations*: *LBD* Lewy body dementia, including both Dementia with Lewy bodies (DLB) and Parkinson’s disease dementia (PDD), Global impression of functional change comprised individuals who got better or worse in their global functioning status. *CI* Confidence interval, *SveDem* the Swedish Registry for Cognitive/Dementia Disorders

The NACC validation cohort partly replicated the patterns observed in SveDem (Table 4). Anchor-based MCIDs were larger in absolute magnitude (LBD: 3.0 points, 95% CI: 2.3 to 3.8; FTD: 4.7 points, 95% CI: 4.0 to 5.3), and again increased with greater baseline impairment, particularly in FTD. Conversely, distribution-based MCIDs in NACC were similar to those in SveDem, generally ranging from 2 to 3 points across diagnoses and strata. Together, these results indicate consistent, diagnosis- and severity-dependent anchor-based MCIDs, alongside relatively stable distribution-based thresholds of 2–3 MMSE points.

**Table 4:** MCID in MMSE in NACC across diagnosis, age, and cognitive function at baseline. Anchor-based MCID values are presented as absolute mean MMSE changes; therefore, larger values indicate greater magnitude of clinically meaningful change, regardless of direction

Diagnosis	N, completed	Change in global status, n (%)	Anchor-based MCID, absolute mean change (95% CI)	Distribution-based MCID
**LBD**				
All individuals	469	149 (31.8)	3.0 (2.3, 3.8)	2.6
Age groups				
≤ 74 y	264	79 (30.0)	3.5 (2.3, 4.6)	2.4
≥ 75 y	205	70 (34.1)	2.5 (1.6, 3.4)	2.9
*Baseline cognition*				
MMSE 15–27	305	118 (38.7)	3.8 (2.9, 4.6)	1.9
MMSE ≥ 26	265	65 (24.5)	2.4 (1.5, 3.2)	0.7
MMSE 20–25	144	58 (40.3)	3.5 (2.2, 4.9)	0.8
MMSE ≤ 19	60	26 (43.3)	3.4 (1.2, 5.7)	2.8
**FTD**				
All individuals	591	255 (43.1)	4.7 (4.0, 5.3)	3.3
Age groups				
≤ 74 y	479	206 (43.0)	4.7 (4.0, 5.5)	3.3
≥ 75 y	112	49 (43.8)	4.5 (3.1, 5.9)	3.3
*Baseline cognition*				
MMSE 15–27	407	186 (45.7)	5.7 (5.0, 6.5)	1.9
MMSE ≥ 26	267	107 (40.1)	3.2 (2.2, 4.2)	0.7
MMSE 20–25	184	79 (42.9)	5.7 (4.4, 6.9)	0.8
MMSE ≤ 19	140	69 (49.3)	5.9 (4.8, 7.0)	3.0

*Abbreviations*: *LBD* Lewy body dementia, including both dementia with Lewy bodies (DLB) and Parkinson’s disease dementia (PDD), *MMSE* Mini-Mental State Examination; Change in global status, comprises individuals who got better or worse in their global functioning status, *CI* Confidence interval, *NACC* the National Alzheimer’s Coordinating Center

### Sensitivity analysis

Sensitivity analyses were conducted within the SveDem LBD cohort, restricting the sample to individuals with DLB or PDD, and analyzing these subgroups separately (Supplementary Table 1). MMSE test–retest reliability and RWRT estimates were highly comparable across subgroups. Within the MMSE ± 2 SD sample, ICCs were 0.8 (95% CI, 0.8‒0.8) in both DLB and PDD, corresponding to RWRTs of 5.2 and 4.7 MMSE points, respectively (Supplementary Table 2). Restricting the baseline MMSE range to 15–27 resulted in lower ICCs—0.72 (95% CI, 0.66‒0.76) for DLB and 0.8 (95% CI, 0.7‒0.8) for PDD—with similar RWRTs of 5.3 and 4.8 points. When all MMSE scores were included, ICCs were 0.7 (95% CI, 0.7‒0.8) for DLB and 0.8 (95% CI, 0.7‒0.8) for PDD, and RWRTs increased to 6.7 and 6.0 points, indicating that changes of approximately 5–7 MMSE points were required to exceed real-world variability in both subgroups (Supplementary Table 3).

MCID sensitivity analyses likewise showed closely aligned estimates for DLB and PDD (Supplementary Table 3). Overall anchor-based MCID was 1.1 MMSE points (95% CI, 0.4‒1.7) in PDD and 0.6 points (95% CI, 0.1‒1.1) in DLB, with corresponding distribution-based MCIDs of 2.1 and 2.2 points. Age-stratified values were similar within each diagnosis, with anchor-based MCIDs around 0.8–1.3 points in PDD and 0.5–0.6 points in DLB, and distribution-based estimates near 2 points. Across baseline MMSE strata, anchor-based MCID was small or negative in the mildest group (MMSE ≥ 26) and increased with greater impairment, reaching 3.2 points (95% CI, 2.2‒4.3) in PDD and 3.3 points (95% CI, 2.4‒4.2) in DLB for MMSE ≤ 19. Distribution-based MCIDs remained relatively stable across strata (~ 1.5–2.2 points), reinforcing the robustness of the LBD thresholds across DLB and PDD subgroups (Supplementary Table 3).

## Discussion

This study provides the first diagnosis-specific estimates of RWRT and MCID for the MMSE in Lewy body dementia (LBD) and frontotemporal dementia (FTD). The estimates were derived from a large national Swedish registry (SveDem) and validated in an independent U.S. cohort (NACC). Across diagnoses, age groups, and cognitive strata, RWRT consistently indicated that a change of 5–7 MMSE points (corresponding to ~ 17–23% of the total MMSE score) was required to exceed expected real-world variability over a 91–400-day interval, with estimates reflecting both measurement variability and true cognitive decline occurring during this period. The RWRT should therefore be interpreted as an interval-specific, real-world threshold for reliable change rather than short-term test–retest error. Distribution-based MCIDs, calculated using the statistical properties of the MMSE, were consistent at 2–3 MMSE points across groups: these values can be interpreted as statistical proxies for important change, reflecting change beyond expected score variability. Anchor-based MCIDs, which are tied to a physician evaluation of clinically meaningful change, differed by diagnosis and baseline severity: they were small in LBD but larger in FTD, particularly at lower MMSE levels. This suggests that small MMSE changes may be clinically meaningful in LBD, whereas larger changes are required to indicate meaningful progression in FTD. All key findings were replicated in NACC, supporting their generalizability. Together, these results provide clinicians and researchers with practical benchmarks for interpreting meaningful cognitive change in LBD and FTD.

Our findings showed that RWRTs of 5–7 MMSE points over the 91–400-day interval were required to exceed the combined variability observed in repeated real-world MMSE assessments. These thresholds should not be interpreted as pure measurement-error estimates, because the follow-up interval was long enough for genuine cognitive decline or fluctuation to occur. These estimates were higher than those reported by Hörnsten et al. [32], who found a smallest detectable change of 4 points over 1–6 days among nursing home residents with Alzheimer’s or vascular dementia, and by Lee et al. [33] who reported high short-term test–retest reliability (ICC ~ 0.6–0.9) with RWRT well below one point over two weeks. These discrepancies reflect differences in cohort characteristics, including age, setting, and dementia type, as well as the longer follow-up interval in our study. Moreover, the reliability of cognitive assessments is not static, and real-world variability may increase over extended periods [34]. Accordingly, longitudinal studies may over- or underestimate cognitive decline if they fail to account for time-dependent variability, and intervention trials risk misclassifying patients as “non-responders” when observed MMSE changes do not exceed interval-specific thresholds for reliable change.

In our study, distribution-based MCIDs varied between approximately 2 and 3 points among individuals with LBD and FTD. To our knowledge, no prior study has reported MCID values for these diagnostic groups. Nevertheless, our findings align with research in individuals with AD [35, 36]. For example, Andrews et al*.* [36] estimated that a 1–3 point MMSE decline corresponded to clinically meaningful deterioration based on clinician-rated change, with MCID values increasing with disease severity. Moreover, a recent review by Muir et al. [35] reported comparable thresholds across AD stages. These observations suggest that the MMSE has stable inherent measurement characteristics despite differences in underlying pathology, and that the magnitude of change required to exceed background variability is relatively consistent across diagnostic groups. This suggests that the sensitivity of the MMSE to meaningful cognitive change is driven primarily by its intrinsic psychometric properties, with additional influence from etiology-specific factors. However, the absence of strong etiology-specific patterns may also reflect limitations in how well the MMSE captures the core clinical features associated with each dementia subtype [37].

Distribution-based MCIDs provided a quantifiable threshold for change but do not inherently capture clinical relevance, which requires clinician judgment or patient-centered anchors to determine meaningfulness in real-world settings. Therefore, we additionally calculated anchor-based MCIDs, which varied by diagnosis, disease severity, and cohort (SveDem versus NACC). Overall, anchor-based MCIDs were smaller among individuals with LBD than among those with FTD, and increased with greater cognitive impairment. Similar severity gradients have been described in AD, with larger changes occurring at more advanced stages [35]. Differences in anchor-based MCID values between SveDem and NACC likely reflect, at least in part, the use of different clinical anchors, whereas discrepancies between diagnostic groups may partly reflect underlying etiological differences across dementias.

To further examine the robustness of our LBD-specific thresholds, we conducted sensitivity analyses separating DLB and PDD, which are often conceptualized as points along an LBD continuum but may differ in clinical profile, motor burden, and diagnostic pathways [38]. Baseline characteristics of the two subgroups were broadly similar, and RWRT and MCID estimates were consistent across DLB and PDD, regardless of whether the analyses were restricted to the MMSE ± 2 SD sample, the 15–27 range, or the full score distribution. In both subgroups, RWRTs were 5–7 MMSE points, and distribution-based MCIDs clustered around ~ 2 points, consistent with the primary LBD analyses. Anchor-based MCIDs also showed comparable patterns, with small values in the mildest strata (MMSE ≥ 26) and increasing magnitudes in more severely impaired participants (MMSE ≤ 19). Collectively, these findings suggest that MMSE measurement properties and clinically meaningful thresholds are largely similar between DLB and PDD in this real-world setting.

Our findings suggest that small MMSE changes should be interpreted cautiously. A 2–3-point change may be meaningful when evaluated at the group level or when supported by external clinical anchors, but it may still fall below the RWRT and therefore should not automatically be interpreted as reliable deterioration in an individual patient. In contrast, a decline of approximately 5–7 MMSE points over 91–400 days is more likely to exceed expected real-world variability, including both retest variability and expected cognitive decline over the observed follow-up interval. Such changes therefore provide stronger evidence of reliable individual-level cognitive change. MCID and RWRT should consequently be viewed as complementary rather than interchangeable: MCID estimates whether a change is potentially meaningful, whereas RWRT estimates whether a change is sufficiently large to be reliable for an individual patient under real-world follow-up conditions. The clinical message is not to overinterpret a 2-point MMSE drop in one patient, especially over variable real-world follow-up intervals, but also not to dismiss 2–3 point group-level changes in studies, because group-level averaging reduces noise and can detect clinically relevant shifts that are not reliable enough for individual decision-making.

Interpreting MMSE change in LBD and FTD must be interpreted against the broader limitations of the MMSE across dementia subtypes [37]. The MMSE is heavily weighted toward orientation and simple memory, with limited assessment of language and visuospatial skills and minimal coverage of executive or social cognition [39]. As Burrell and Piguet describe [39], the MMSE is relatively insensitive to the executive, behavioral, and language impairments that characterize FTD, and to the visuospatial and attentional deficits and fluctuating cognition typical of LBD, such that substantial syndrome-specific deterioration may occur “off-axis” relative to what the MMSE measures. Bruun et al. [37] further highlighted that although the MMSE and related cognitive tests reliably distinguish dementia from controls, they perform poorly in differentiating AD, LBD, and FTD. Consequently, our diagnosis-specific RWRT and MCID estimates underscore a key methodological challenge: clinicians and researchers must often interpret small MMSE changes in disorders where core cognitive and behavioral features lie outside the test’s principal domains, increasing the risk of underestimating or misclassifying disease-specific progression in LBD and FTD or relying on thresholds derived primarily from AD-focused cohorts [37]. With this in mind, future studies should evaluate domain-specific and digital measures that may better capture executive, language, visuospatial, and fluctuating cognitive changes in FTD and LBD. Finally, because the RWRT was derived from assessments separated by 91–400 days, the estimates may partly incorporate true cognitive change in addition to real-world variability. This may have reduced ICC values and inflated RWRT estimates compared with short-term test–retest studies. The reported RWRT values should therefore be regarded as conservative thresholds for reliable annual MMSE change in real-world settings.

To our knowledge, this study is the first to report diagnosis-specific RWRT and MCID values for the MMSE in LBD (including DLB and PDD) and FTD using two large, independent cohorts from Sweden and the United States. The use of harmonized analytic procedures across SveDem and NACC, together with both distribution- and anchor-based approaches, aligns with previously described methods [40] and enhances the comparability and external validity of our findings.

### Limitations

This study has some limitations. SveDem is a clinical quality registry with an estimated coverage of approximately 29% [20]; the extent to which individuals are representative of the broader LBD and FTD populations is uncertain, raising the possibility of selection bias. Conversely, NACC is a research-oriented database that is not representative of clinical populations; accordingly, the two cohorts differ in recruitment context, case mix, and follow-up procedures. Diagnostic criteria and their clinical application may also vary between SveDem and NACC, potentially introducing misclassification and limiting direct comparability. In our study, FTD was analyzed as a single diagnostic group because subtype information was not consistently available in SveDem. Given the clinical heterogeneity of FTD, including behavioral and language-led phenotypes, the reported FTD thresholds should be interpreted as overall estimates.

Different clinical anchors were used to estimate the MCID (the global impression of functional change in SveDem versus CDR change in NACC), complicating the transferability of anchor-based thresholds between cohorts and suggesting that such estimates may not generalize to populations with different characteristics or anchors. In addition, although MMSE administration was performed by trained clinicians, the large number of raters across settings may have introduced interrater variability or deviations from standardized administration. Moreover, as this was a retrospective registry-based study, we could not control the exact conditions under which the MMSE was administered. These uncontrolled factors may have contributed to variability in repeated MMSE scores and may have increased the estimated RWRT. Therefore, the reported thresholds should be interpreted as reflecting reliable change under routine clinical conditions, rather than under tightly standardized research conditions. Because the RWRT was derived from assessments separated by 91–400 days, the estimates most likely incorporated true cognitive change in addition to measurement variability.

Many individuals were not included in the cohorts because they lacked MMSE follow-up within the prespecified 91–400-day interval. Although this reflects the eligibility criteria, it may limit representativeness. The included SveDem cohort was slightly younger and had a higher proportion of men than those excluded, which may further limit the generalizability of the RWRT and MCID estimates. Differences in age and sex may be associated with variation in disease progression rates, health-seeking behaviors, and retention patterns, as well as with the longitudinal stability of MMSE performance. Consequently, the reported thresholds should be interpreted with caution when applied to older or more female-dominated clinical samples.

## Conclusions

Over a follow-up interval of 90–400 days, a change of approximately 5–7 MMSE points was needed to exceed expected variability and expected cognitive decline during the follow-up period of 91–400 days. These thresholds therefore do not represent short-term measurement error alone, but rather conservative real-world thresholds for reliable individual-level change over approximately one year. At the same time, smaller changes of approximately 2–3 MMSE points may still be clinically meaningful, particularly at the group level or when supported by patient-reported or clinician-rated functional change. MCID and RWRT should therefore be used together: MCID helps identify potentially meaningful change, whereas RWRT helps determine whether the change is large enough to be considered reliable in an individual patient. Clinically, this means that a 2-point MMSE decline in a single patient should not automatically be interpreted as definite progression, especially over variable real-world follow-up intervals. However, similar changes at the group level may still be important in observational studies or clinical trials. These findings provide practical, diagnosis-specific benchmarks for interpreting MMSE change in LBD and FTD, while also emphasizing the need to consider clinical context, disease stage, and the known limitations of the MMSE in capturing the core symptoms of these disorders.

## Supplementary Information

Supplementary Material 1.

## Data Availability

No datasets were generated or analysed during the current study.

## Abbreviations

AD: Alzheimer’s disease
ADRC: Alzheimer’s Disease Research Center
CI: Confidence interval
CDR: Clinical Dementia Rating
CDR® Plus NACC FTLD: Clinical Dementia Rating Plus NACC Frontotemporal Lobar Degeneration Behaviour & Language Domains
DLB: Dementia with Lewy bodies
FTD: Frontotemporal dementia
ICC: Intraclass correlation coefficient
IQR: Interquartile range
LBD: Lewy body dementia
LoA: Limits of agreement
MCID: Minimum clinically important difference
MDC: Minimal detectable change
MMSE: Mini-Mental State Examination
NACC: National Alzheimer’s Coordinating Center
PDD: Parkinson’s disease dementia
RWRT: Real-World Reassessment Threshold
SD: Standard deviation
SveDem: Swedish Registry for Cognitive/Dementia Disorders
UDS: Uniform Data Set
