# Supplementary material for: Thresholds for meaningful change in Mini-Mental State Examination scores in rare dementias
Source: Alzheimers Res Ther. 2026 Jul 11;18:165. doi: 10.1186/s13195-026-02136-y (PMC13366843; doi:10.1186/s13195-026-02136-y)
Supplement: Supplementary file 1 — Supplementary Material 1. [file 13195_2026_2136_MOESM1_ESM.docx]

# Online supplement


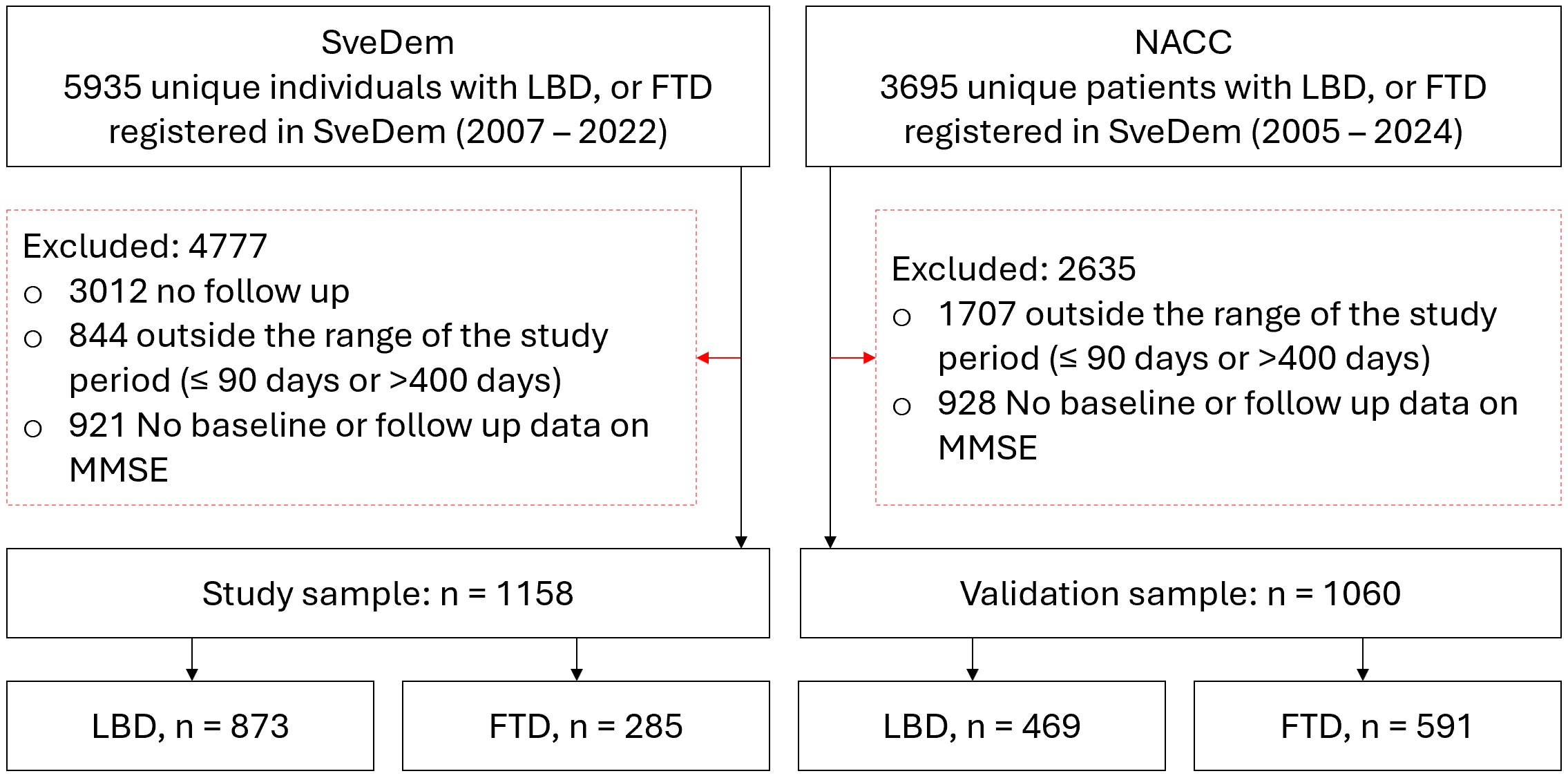


Supplementary Figure 1. Flowchart of participant inclusion and exclusion. Abbreviations: SveDem, the Swedish Registry for Cognitive/Dementia Disorders; NACC, National Alzheimer’s Coordinating Center; LBD Lewy Body Dementia included individuals with Parkinson’s disease dementia or Dementia with Lewy Bodies; FTD, Frontotemporal dementia; MMSE, Mini-Mental State Examination.

Supplementary Table 1. Baseline demographic of individuals with Dementia with Lewy Bodies (DLB) and Parkinson's Disease Dementia (PDD), SveDem data.

|  | DLB, n = 583 | | PDD, n = 290 | |
| --- | --- | --- | --- | --- |
| Variables/categories | Mean (SD) or n (%) | 95% CI | Mean (SD) or n (%) | 95% CI |
| Age at diagnosis | 75.2 (6.2) | 74.7, 75.7 | 74.3 (6.9) | 73.5, 75.1 |
| **Sex** |  |  |  |  |
| Male | 395 (67.8) | 63.9, 71.5 | 198 (68.3) | 62.8, 73.4 |
| Female | 188 (32.2) | 28.5, 36.1 | 92 (31.7) | 26.6, 37.2 |
| **Education** |  |  |  |  |
| Primary or below (≤ 9 y) | 55 (23.1) | 18.1, 28.8 | 25 (22.5) | 15.5, 30.9 |
| Secondary (10 - 12 y) | 99 (41.6) | 35.5, 47.9 | 46 (41.4) | 32.6, 50.7 |
| University or above (≥13 y) | 84 (35.3) | 29.4, 41.5 | 40 (36.0) | 27.6, 45.2 |
| **Marital status** |  |  |  |  |
| Married/cohabiting | 160 (67.2) | 61.1, 73.0 | 71 (63.4) | 54.2, 71.9 |
| Widowed | 23 (9.7) | 6.4, 13.9 | 11 (9.8) | 5.3, 16.4 |
| Single/divorced | 55 (23.1) | 18.1, 28.8 | 30 (26.8) | 19.3, 35.5 |
| **Cohabitation status** |  |  |  |  |
| Living alone | 126 (25.6) | 21.9, 29.6 | 40 (16.2) | 12.0, 21.2 |
| Cohabiting/other | 366 (74.4) | 70.4, 78.1 | 207 (83.8) | 78.8, 88.0 |
| **Accommodation** |  |  |  |  |
| Ordinary housing | 513 (88.6) | 85.8, 91.0 | 249 (85.9) | 81.5, 89.5 |
| Institutional care | 66 (11.4) | 9.0, 14.2 | 41 (14.1) | 10.5, 18.5 |
| **Baseline cognition** |  |  |  |  |
| MMSE ≥ 26 | 146 (25.0) | 21.7, 28.7 | 54 (18.6) | 14.5, 23.4 |
| MMSE 20–25 | 279 (47.9) | 43.8, 51.9 | 144 (49.7) | 43.9, 55.4 |
| MMSE ≤ 19 | 158 (27.1) | 23.6, 30.8 | 92 (31.7) | 26.6, 37.2 |
| Abbreviations: MMSE, Mini-Mental State Examination; SD, standard deviation; 95% CI, 95% confidence interval. SveDem, the Swedish Registry for Cognitive/Dementia Disorders. | | | | |

Supplementary Table 2. Sensitivity analysis of Real-Word Reassessment Threshold (RWRT) in MMSE among individuals with Dementia with Lewy Bodies (DLB) and Parkinson’s disease dementia (PDD) subgroups from SveDem.

|  | **DLB** | **PDD** |
| --- | --- | --- |
| **MMSE, within ±2 SD** |  |  |
| MMSE baseline, mean (95 % CI) | 22.5 (22.2, 22.9) | 21.9 (21.4, 22.4) |
| MMSE follow-up, mean (95 % CI) | 22.3 (21.9, 22.6) | 21.5 (21.0, 22.0) |
| SD baseline | 4.1 | 4.1 |
| SD follow-up | 4.3 | 4.3 |
| SD pooled | 4.2 | 4.2 |
| ICC (95 % CI) | 0.8 (0.8, 0.8) | 0.8 (0.8, 0.9) |
| RWRT | 5.2 | 4.7 |
| **MMSE 15–27 at baseline** |  |  |
| MMSE baseline, mean (95 % CI) | 22.3 (22.0, 22.5) | 22.1 (21.7, 22.5) |
| MMSE follow-up, mean (95 % CI) | 22.0 (21.7, 22.4) | 21.7 (21.2, 22.2) |
| SD baseline | 3.2 | 3.3 |
| SD follow-up | 4.0 | 3.8 |
| SD pooled | 3.6 | 3.5 |
| ICC (95 % CI) | 0.7 (0.7, 0.8) | 0.8 (0.7, 0.8) |
| RWRT | 5.3 | 4.8 |
| **All MMSE scores** |  |  |
| MMSE baseline, mean (95 % CI) | 22.4 (22.1, 22.8) | 21.9 (21.4, 22.4) |
| MMSE follow-up, mean (95 % CI) | 22.1 (21.7, 22.4) | 21.3 (20.8, 21.8) |
| SD baseline | 4.3 | 4.2 |
| SD follow-up | 4.6 | 4.5 |
| SD pooled | 4.5 | 4.4 |
| ICC (95 % CI) | 0.7 (0.7, 0.8) | 0.8 (0.7, 0.8) |
| RWRT | 6.7 | 6.0 |
| Abbreviations: MMSE, Mini-Mental State Examination; SD, standard deviation; ICC (95% CI), intraclass correlation coefficient with 95% confidence interval; RWRT, real-word reassessment threshold at the 95% confidence level. For the “MMSE change within ±2 SD” analysis, outliers were removed using a ±2 SD rule on the change score within each diagnostic group. The other analyses include the specified baseline MMSE range or all available MMSE scores; SveDem, the Swedish Registry for Cognitive/Dementia Disorders. | | |

Supplementary Table 3. Sensitivity analysis on Minimal Clinically Important Difference in MMSE In SveDem: anchor and distribution estimates across Dementia with Lewy bodies (DLB) and Parkinson’s disease dementia (PDD) based on age, and cognitive function strata at baseline.

| Diagnosis | N, completed | Change in global status  n (%) | Anchor-based MCID, absolute mean change (95% CI) | Distribution-based MCID |
| --- | --- | --- | --- | --- |
| PDD |  |  |  |  |
| All participants | 290 | 164 (56.6) | 1.1 (0.4, 1.7) | 2.1 |
| Age groups |  |  |  |  |
| ≤ 74 | 143 | 81 (56.6) | 0.8 (0.2, 1.9) | 2.2 |
| ≥ 75 | 147 | 83 (56.5) | 1.3 (0.4, 2.2) | 2.0 |
| Baseline cognition |  |  |  |  |
| MMSE 15–27 | 254 | 139 (54.7) | 0.7 (0.0, 1.3) | 1.9 |
| MMSE ≥ 26 | 54 | 29 (53.7) | 2.6 (1.3, 3.8) | 1.5 |
| MMSE 20 – 25 | 144 | 69 (47.9) | 0.5 (0.4, 1.4) | 1.6 |
| MMSE ≤ 19 | 92 | 66 (71.7) | 3.2 (2.2, 4.3) | 2.1 |
| DLB |  |  |  |  |
| All participants | 583 | 354 (60.7) | 0.6 (0.1, 1.1) | 2.2 |
| Age groups |  |  |  |  |
| ≤ 74 | 254 | 151 (59.4) | 0.5 (0.2, 1.3) | 2.2 |
| ≥ 75 | 329 | 203 (61.7) | 0.6 (0.1, 1.3) | 2.1 |
| Baseline cognition |  |  |  |  |
| MMSE 15–27 | 494 | 294 (59.5) | 0.5 (0.0, 1.0) | 2.0 |
| MMSE ≥ 26 | 146 | 72 (49.3) | 2.9 (2.1, 3.7) | 1.5 |
| MMSE 20 – 25 | 279 | 168 (60.2) | 0.2 (-0.4, 0.8) | 1.8 |
| MMSE ≤ 19 | 158 | 114 (72.2) | 3.3 (2.4, 4.2) | 2.2 |
| Abbreviations: CI, confidence interval; DLB, dementia with Lewy bodies; MCID, minimum clinically important difference; MMSE, Mini-Mental State Examination; PDD, Parkinson’s disease dementia; SveDem, Swedish Registry for Cognitive/Dementia Disorders. Change in global functioning status comprises individuals classified as improved or worsened in global functioning. Anchor-based MCID values are presented as absolute mean MMSE changes; therefore, larger values indicate greater magnitude of clinically meaningful change, regardless of direction. Anchor-based MCID values were calculated among participants with available anchor data and MMSE data. | | | | |
